# Impact of Sample Preservation and Manipulation on Insect Gut Microbiome Profiling. A Test Case With Fruit Flies (Diptera, Tephritidae)

**DOI:** 10.3389/fmicb.2019.02833

**Published:** 2019-12-13

**Authors:** Maarten De Cock, Massimiliano Virgilio, Peter Vandamme, Antonios Augustinos, Kostas Bourtzis, Anne Willems, Marc De Meyer

**Affiliations:** ^1^Department of Biology and Joint Experimental Molecular Unit, Royal Museum for Central Africa, Tervuren, Belgium; ^2^Laboratory of Microbiology, Faculty of Sciences, Ghent University, Ghent, Belgium; ^3^Department of Plant Protection, Institute of Industrial and Forage Crops, Hellenic Agricultural Organization – Demeter, Patras, Greece; ^4^Insect Pest Control Laboratory, Joint FAO/IAEA Programme of Nuclear Techniques in Food and Agriculture, Vienna, Austria

**Keywords:** amplicon sequencing, gut microbiome, methodology, Tephritidae, *Ceratitis capitata*

## Abstract

High-throughput sequencing (HTS) techniques are of great value for the investigation of microbial communities, and have been extensively used to study the gut microbiome. While most studies focus on the human gut, many others have investigated insects. However, because of the rapid spread of HTS techniques, a lot of variation exists in the protocols for sample preparation. In the present study, we investigated the impact of two widely adopted sample-processing procedures preceding library preparation, i.e., preservation of insect tissue in 70% ethanol (EtOH) and sample dissection. We used the fruit fly *Ceratitis capitata* (Diptera: Tephritidae) as a model organism and set up two experiments, one comparing the effects of sample manipulation and preservation across life stages and the other across fruit samples from different sources. The results of this study showed no major effects of dissection on the outcome of HTS. However, EtOH preservation did have effects on the recovered gut microbiome, the main effect being a significant reduction of the dominant genus, *Providencia*, in EtOH-preserved samples. Less abundant bacterial groups were also affected resulting in altered microbial profiles obtained from samples preserved in 70% EtOH. These results have important implications for the planning of future studies and when comparing studies that used different sample preparation protocols.

## Introduction

Microbial communities are an integral part of the functioning and survival of all ecosystems and all living organisms (Holguin et al., 2001; Rosenberg et al., 2007; Zilber-Rosenberg and Rosenberg, 2008). In recent decades, the emergence of high-throughput sequencing (HTS) techniques has revolutionized the study of these communities (Metzker, 2010; Morey et al., 2013). These methods allow large amounts of information about microbiological communities to be collected in a relatively short time, without the need of specialized microbiological techniques. However, the fast emergence of this technique led to a large diversity of protocols applied.

Using HTS techniques, increasing amounts of information have been collected describing the gut microbiome and its potential benefits to the host fitness. While a lot of this research has been focused on the human gut microbiome (NIH HMP Working Group et al., 2009; Kinross et al., 2011), several studies have addressed other organisms, including insects (Dillon and Dillon, 2004; Alma, 2008; Engel and Moran, 2013; Raymann and Moran, 2018). These studies have linked microbiological activity to improved digestion of indigestible components, including toxins, increased reproductive output, and many other factors benefiting the host. However, recent studies have indicated potential large effects of different sample preparation protocols in the study of microbiological communities (Hale et al., 2015; Hammer et al., 2015; Song et al., 2016). In fecal samples, one of the most crucial aspect emerging from these studies is preservation, as multiple studies have reported large effects of different preservation protocols on the microbiological community (Vlčková et al., 2012; Hale et al., 2015; Song et al., 2016). Therefore, the use of a strictly standardized protocol for sample processing is of utmost importance.

Tephritid fruit flies (Diptera: Tephritidae) are well-known pests in agricultural- and horticultural crops. Worldwide there are more than 4600 species of fruit flies (White and Elson-Harris, 1992; Uchôa, 2014), of which a large part utilizes fruits for larval development, as suggested by their name. This causes enormous losses, both directly, by damaging the fruit tissue, and indirectly, by accelerating the rotting process and infestation by other insects, fungi, and bacteria. Because of this, infestations by fruit flies can have huge economic impacts on the agricultural sector. The Mediterranean fruit fly (Medfly), *Ceratitis capitata*, is one of the most wide spread and notorious fruit fly species (De Meyer et al., 2008; Dominiak and Daniels, 2012). While native to sub-Saharan Africa, it has spread throughout the Mediterranean region, Latin-America, and Western Australia, with occasional records from North-America (De Meyer et al., 2008). One of the main reasons behind this widespread occurrence is its highly polyphagous nature, enabling it to exploit a wide variety of plant species. The host species can thus vary depending on the available plant species in a particular region or time. Currently, *C. capitata* infestations have been found in more than 260 plant species. Many of these host species are agricultural crops, and therefore the control of these species is of uttermost importance. The gut microbiome of tephritid fruit flies has increasingly been studied in the recent years, revealing the presence and role of many microorganisms. Examples include bacteria helping to overcome pesticides (Cheng et al., 2017) and host defenses (Ben-yosef et al., 2015) or generally increasing longevity of fruit flies (Behar et al., 2008b). Nevertheless, the overall knowledge of the fruit fly microbiome is still very fragmented and lacking in many areas.

In the study of the insect gut microbiome variation in the preparation protocol has only been studied to a limited extent. Although, as for fecal samples, we can suspect effects of the choice of preparation protocol in this kind of samples, empirical evidence for this is still lacking. In the present study, we set out to analyze two aspects of sample processing for HTS study of the gut microbiome of insects, using *C. capitata* (Diptera: Tephritidae) as a model organism. The first aspect of the sample processing protocol that was analyzed is the effect of dissection of the gut from the insect body. The removal of the gut (or other insect organs) is a well-established part of the protocol of many gut microbiome studies (Husseneder and Grace, 2005; Ami et al., 2010; Gavriel et al., 2011; Colman et al., 2012; Augustinos et al., 2015; Clarke, 2016; Zhang et al., 2016). However, since dissecting the gut from bodies of tiny insects, such as fruit flies, is challenging and time consuming, this step is often omitted from the sample preparation protocol (Wong et al., 2011; Ceja-Navarro et al., 2015; Garofalo et al., 2017). Although dissection might have a large impact on the gut microbiome assemblage recovered, to our knowledge, no studies have previously assessed this effect. Our hypothesis is that dissection in fruit flies, and in particular larvae, has no significant effect on the gut microbiome profiles recovered through HTS and can therefore be omitted from the protocol. The second aspect that was studied is the effect of storing individual insects in 70% ethanol (EtOH). As EtOH is a product that is easily acquired and transported, it is ideal for the collection and storage of fruit flies, even in countries where fast cooling is less evident. Additionally, preserving insects in 70% EtOH has the benefit of keeping insects flexible enough to make manipulations, such as removal of the gut, feasible, while this is not actually possible with specimens preserved in 100% EtOH, which become more fragile and tend to break during dissection. In fecal samples, 70% EtOH has been reported to have some effect on the microbiome recovered and its use is advised against (Vlčková et al., 2012; Hale et al., 2015; Song et al., 2016). Therefore, identifying and quantifying how EtOH preservation affects the gut microbiome in insect bodies should provide valuable information. Our hypothesis is that preserving insect samples in 70% EtOH for long periods will have no major effects on the gut microbiota and major patterns in microbiome composition will still emerge even after preservation.

Like many insects, tephritid fruit flies go through a significant metamorphosis during their development to adults. It has been shown in previous studies that this metamorphosis in insects, including tephritid fruit flies, can have major effects on the gut microbiome (Morales-Jiménez et al., 2012; Aharon et al., 2013; Andongma et al., 2015). Additionally, recent studies have shown that there are major differences in the gut microbiome between different populations within the same fruit fly species (Wang et al., 2011, 2014). In the present study, we aim at verifying if widely adopted insect preservation and manipulation procedures might significantly bias the HTS profiling of their gut microbial communities. This will provide important baseline information to interpret and compare data from different studies.

## Materials and Methods

### Experimental Setup

In a first experiment, we investigated the effects of gut dissection and sample preservation across different life stages of *C. capitata*. Reared specimens were provided by the Insect Pest Control Laboratory (IPCL) of the Joint FAO/IAEA Division of Nuclear Techniques in Food and Agriculture, International Atomic Energy Agency (IAEA). We sampled third-instar larvae, tenerals (i.e., newly emerged adults) and mature adults from a long-established laboratory colony population (>30 years; >400 generations) of Greek origin. More than 60 individuals were collected for each life stage, 30 of which were processed immediately (see below) and the remaining individuals were stored in 70% EtOH at −20°C for a period varying from 12 to 18 weeks.

In a second experiment we evaluated the effects of sample dissection across EtOH-preserved third-instar larvae from different sources. We sampled three different laboratory colonies of *C. capitata*, provided by the IPCL. This included the long-established colony population (>30 years; >400 generations) of Greek origin, an intermediate established colony population (eight generations) of Australian origin and a newly established colony population (one generation) of Argentinian origin. Larvae collected from a wild population from Italy were also included in the experimental setup. Before processing the wild population, the identity of each larva was confirmed via DNA barcoding (see Supplementary Table S1) as described in Virgilio et al. (2012). Each sample was composed of more than 30 larvae that were immediately stored in 70% EtOH (see Supplementary Tables S2, S3 for details on the experimental design and sample collection).

### Laboratory Procedures

Before sample processing, the body surface of all insect specimens was sterilized in 70% EtOH for 30 s and then rinsed once in phosphate-buffered saline (PBS) water. Guts were dissected by removing, with sterilized tools, the whole gut from crop to anus. Undissected, full bodies were directly crushed with a sterilized pestle. In order to minimize biases due to inter-individual variability, five dissected guts or crushed bodies were pooled per sample and DNA was extracted from each pool using the Qiagen DNAEasy extraction kit as per the manufacturer’s protocol. Before genomic library preparation, DNA concentrations of samples were determined using a Qubit Fluorometer (Thermofisher). Samples with DNA concentration <1 ng/μl were discarded and DNA extraction repeated on a novel set of specimens. The genomic library preparation targeted the V3–V4 region of the 16S ribosomal RNA gene [rRNA, insert size 465 bp, 341 F, and 806R primers (Takahashi et al., 2014)] and relied on the Nextera XT kit (including Illumina sequencing adapters, and dual-index barcodes) as per the manufacturer’s protocol. DNA was amplified in two steps and, for most samples, the second amplification was repeated to increase DNA yield. A final quality check of fragment size distributions was performed using an Agilent 2100 Bioanalyzer system. A mock community including DNA of 18 bacterial strains from the BCCM/LMG Bacteria Collection^1^ and a blank were also included in the Illumina run as positive and negative controls, respectively. Libraries were sequenced on an Illumina MiqSeq platform [300 bp paired end (PE) sequencing, performed by Macrogen].

### Analysis of Data

Read quality was preliminarily assessed in FastQC (Andrews, 2014) and data filtering implemented via the DADA2 pipeline (Callahan et al., 2016) in R. This pipeline is based on a self-learning algorithm that compiles a parametric error model fitting the raw data, which is then used to infer sequencing errors. After trimming, demultiplexing, and filtering, paired reads were assigned to operational Taxonomic Units (OTUs) according to the Bayesian classifier method implemented by DADA2 (Wang et al., 2007). Results for the mock community and blanks were used to determine quality of the analysis. The Silva reference database v132 (Pruesse et al., 2012) was used for taxonomic assignment of OTUs (percentage of identity = 97% similarity, p-min-consensus = 0.51) and the robustness of taxonomic assignment was double-checked using the RDP (Cole et al., 2014) and Greengenes databases (DeSantis et al., 2006; data not shown). The complete analytical pipeline is detailed in Supplementary Table S4. Before further analysis singletons and double tons were removed from the data and OTUs with a significant presence in the blanks were deleted. For comparison between samples data scaling, based on the median sample number of reads, was implemented (de Cárcer et al., 2011).

Downstream analyses were done in R, using the Phyloseq (McMurdie and Holmes, 2013) and Vegan (Oksanen et al., 2017) packages. Differences in univariate patterns of alpha diversity [as estimated by the Reverse Simpson index (Lande, 2016)], calculated from OTU data, were tested via analysis of variance (ANOVA), with life stage (larva, teneral, and adult), preservation (fresh vs. EtOH preserved), and dissection (dissected gut vs. full body) as fixed, orthogonal factors for the first experiment and dissection (dissected gut vs. full body) and sample origin (colony strain Greece, colony strain Australia, colony strain Argentina, wild population Italy) as fixed, and random orthogonal factors, respectively, for the second experiment. *A posteriori* pairwise comparisons of significant factors were implemented via Tukey’s honestly significant difference (HSD) test (Abdi and Williams, 2010).

Multivariate differences in OTU abundance and composition were tested using permutational multivariate analysis of variance (PERMANOVA; Anderson, 2017) and permutational multivariate analysis of dispersion (PERMDISP; Anderson, 2006) on scaled data as implemented by the programs PERMANOVA and PERMDISP. For PERMANOVA we relied on the same three-way factorial setup described above for univariate analyses, while for PERMDISP, which only applies to two-ways experimental designs, we tested the effects of life stage and preservation. *A posteriori* pairwise comparisons of multivariate significant factors were then implemented using the permutational *t*-statistics of PERMANOVA and PERMDISP. Probability values of repeated *a posteriori* tests were corrected for Type I errors using the false discovery rate procedure (Benjamini and Hochberg, 1995) with experiment-wise probability *p* = 0.05. Multivariate patterns were visually interpreted via scaled and centered principal coordinates analyses (PCoAs) based on Bray–Curtis distance (Bray and Curtis, 1957) as implemented by the R package ggplot2 (Wickham, 2009).

## Results

### Overall Bacterial Diversity Associated With *C. capitata*

The Illumina Miseq run yielded more than 13 × 10^6^ PE reads across the 54 samples considered. After assessing the quality of the reads in FastQC (Andrews, 2014), forward and reverse reads were trimmed to 230 and 200 bp, respectively. Based on read quality, a strict error rate (max *N*s = 0, max error rate = 1, see Supplementary Table S4) was applied. After quality control, demultiplexing, pairing, and filtering, we obtained a total of 3.6 × 10^6^ reads, corresponding to a total of 848 unique OTUs. Taxonomic assignment of OTUs yielded 155 genera from 13 phyla (Supplementary Table S6). The phylum Proteobacteria was by far the most dominant (91.64% of reads) and comprised a few dominant genera: *Acinetobacter* (19.03%), *Pluralibacter* (11.31%), *Morganella* (7.40%), *Klebsiella* (3.36%), *Serratia* (2.06%), and *Enterobacter* (1.44%). The second most important phylum, Firmicutes (8.21%), included different genera from the orders Bacillales [mainly represented by *Bacillus* (2.15%), *Staphylococcus* (1.77%), and *Salinicoccus* (1.19%)], and Lactobacillales [mainly including *Lactococcus* (1.08%) and *Lactobacillus* (0.78%)]. Genera from the phyla Bacteroidetes (0.44%) and Actinobacteria (0.22%) could also be identified. The remaining phyla represented <0.01% of the total reads. A full overview of the OTU composition can be found in Supplementary Table S5. Comparison between samples used not the total read output as described above but a scaled dataset.

### Impact of Sample Preservation and Manipulation Across Life Stages

**FIGURE 1:**
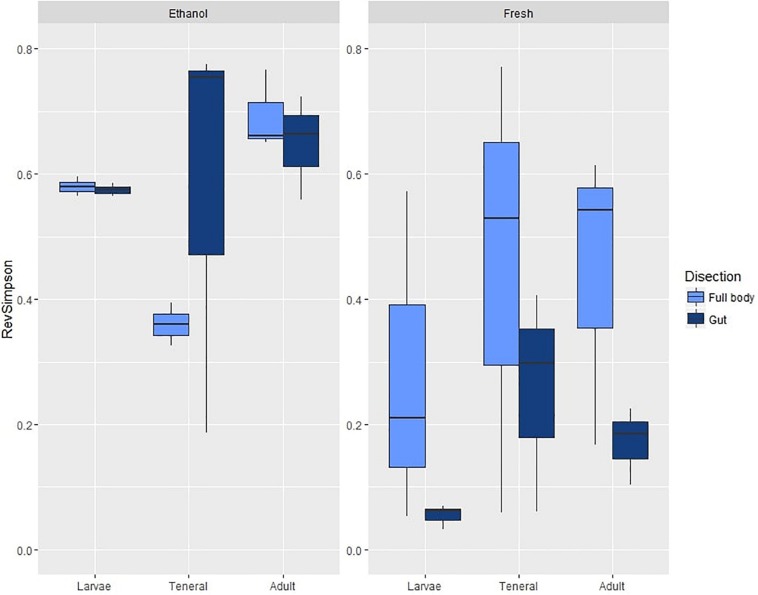
Box plots of OTU diversity (as estimated by the Reverse Simpson index) of microbial assemblages in ethanol preserved/fresh and gut/full body specimens of *C. capitata*. Standard deviations are shown as error bars.

On average, samples from the first experiment had 47.26 OTUs (SD = 28.30) and a reverse Simpson index of 0.43 (SD = 0.26). ANOVA on the reverse Simpson index obtained from the OTU data showed no effects of dissection or life stage on diversity (Table 1A and Supplementary Table S7a). However, a highly significant effect of preservation, with lower diversity in fresh compared to EtOH-preserved samples was found (Figure 1).

**TABLE 1:** Summary table for ANOVA, PERMANOVA, and PERMDISP testing for differences in patterns of alpha diversity (as estimated by the Reverse Simpson index calculated from OTU data) across **(A)** dissection procedures, sample preservation methods, and life stages of *C. capitata* and **(B)** dissection procedures on different populations of *C. capitata*.

	**ANOVA**	**PERMANOVA**	**PERMDISP**
**(A)**			
Life stage (li)	n.s.	*⁣*⁣*	n.s.
Preservation (pr)	*⁣*⁣*	*⁣*⁣*	n.s.
Dissection (di)	n.s.	n.s.	
Li × pr	n.s.	*⁣*⁣*	*⁣*⁣*
Li × di	n.s.	n.s.	
Pr × di	n.s.	*	
Li × pr × di	n.s.	n.s.	
**(B)**			
Origin (or)	n.s.	*⁣*⁣*	*
Dissection (di)	n.s.	n.s.	n.s.
Or × di	n.s.	n.s.	n.s.

*Full tables available in Supplementary Table S7. n.s., not significant; ^∗^, significant at *P* < 0.05, ^∗∗∗^, at *P* < 0.001.*

Permutational multivariate analysis of variance revealed a highly significant interaction of preservation and life stage (at *P* < 0.001) as well as a significant interaction of preservation and dissection (at *P* = 0.047) (Table 1A and Supplementary Table S8a). The *a posteriori* comparisons showed that EtOH preservation always had a significant effect on the multivariate patterns of microbial assemblages. Interestingly, significant differences across life stages were detected across all EtOH-preserved specimens, while in the microbial assemblages of fresh specimens, significant variations were only observed when comparing tenerals to adults (Supplementary Table S8a). Effects of dissection were only detected in fresh samples (*P* = 0.040) (Supplementary Table S8a).

Permutational multivariate analysis of dispersion showed a significant interaction of life stage and preservation (Table 1A and Supplementary Table S9a). Pairwise *a posteriori* tests (Supplementary Table S9a) revealed that the multivariate patterns of dispersion of fresh samples were comparable (with average within group dissimilarities ranging from 14.717 to 26.374) and not significantly different, while in EtOH-preserved samples the multivariate dispersion of tenerals, adults, and larvae significantly differed with average within group dissimilarities of, respectively, 58.335, 31.031, and 2.091.

**FIGURE 2:**
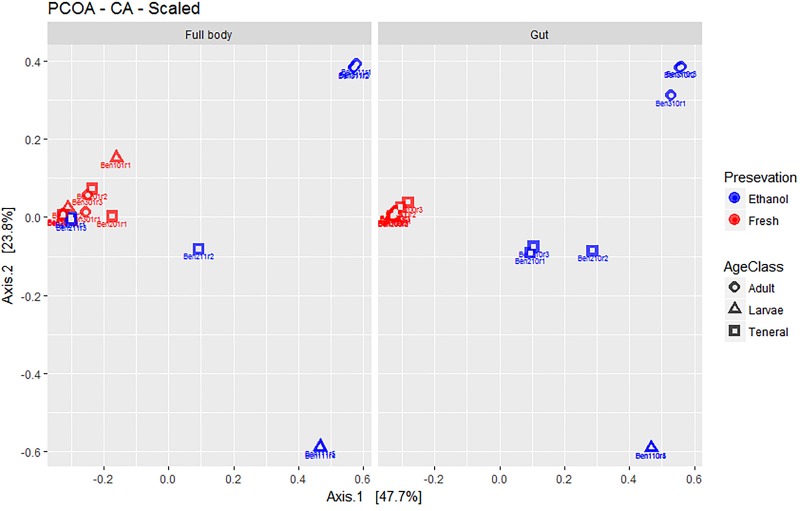
Multivariate ordination (PCOA) of gut microbial assemblages in fresh and ethanol preserved specimens of larvae, teneral, and adults of *C. capitata*. For clarity, the same PCOA is represented twice, either with full body **(left)** or gut-dissected samples **(right)** indicated. Colors refer to preservation (blue: ethanol preserved and red: fresh) and shapes to life stage (circle: adult, triangle: larvae, square: teneral).

Overall, the first two axes of the PCOA (Figure 2) explained 71.9% of variation (47.7 and 23.8% for PC1 and PC2, respectively). The visual inspection of graphs again suggested a lack of major differences between full body and dissected gut samples. EtOH preservation showed distinct differences where fresh individuals of all life stages combined, while EtOH-preserved samples form distinct groups corresponding to their life stage. Fresh samples of tenerals and adults were markedly less dispersed around their group centroid compared to their EtOH-preserved counterparts. Interestingly, fresh larvae have a remarkably higher dispersion compared to EtOH-preserved larvae.

**FIGURE 3:**
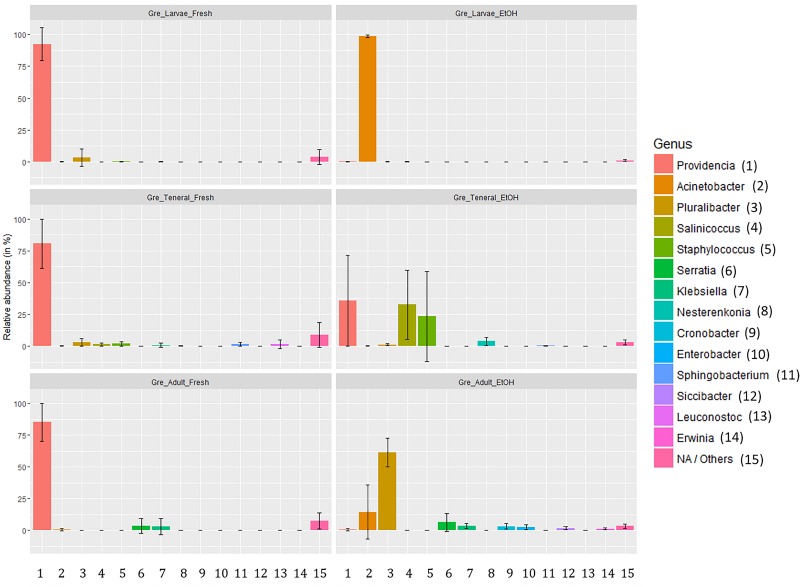
Relative abundance of gut symbionts (genus level classification) in fresh and ethanol preserved specimens of larvae, teneral, and adults of *C. capitata* from the colony strain of Greek origin.

The relative abundance of the most common taxa across treatments did not suggest any obvious effect of dissection in either fresh or EtOH-preserved samples (Supplementary Table S10). However, as indicated by PERMANOVA there were some effects of dissection within fresh samples. In taxon composition this was apparent as a lower dominance of *Providencia* in full body samples (gut: 93.84%, SD: 7.00%; full: 78.46%, SD: 18.66%). Conversely, there was a slightly higher relative abundance of many other genera in full body samples. However, there were only two genera, *Serratia* and *Klebsiella*, where this difference exceeded 1% relative abundance. Conversely, EtOH preservation heavily affected the composition of the gut microbiome with not consistent effects from life stage to life stage (Figure 3). Overall, we could observe a general trend from dominance of *Providencia* in fresh samples (larvae: 92.57%, SD: 12.81%; tenerals: 80.66%, SD: 19.27%; adults: 85.22%, SD: 15.04%) to a strong decline of *Providencia* (larvae: 0.14%, SD: 0.19%; tenerals: 35.83%, SD: 35.82%; adults: 0.90%, SD: 0.96%) and dominance of one or more other genera in EtOH-preserved samples. In EtOH-preserved larvae, we observed *Acinetobacter* as the dominant genus (fresh: 0.05%, SD: 0.05%; EtOH: 98.63%, SD: 1.11%). In EtOH-preserved tenerals, *Providencia* (fresh: 80.66%, 19.27%; EtOH: 35.83%, SD: 35.83%), *Salinicoccus* (fresh: 1.35%, SD: 1.1%; EtOH: 32.60%, SD: 27.23%), and *Staphylococcus* (fresh: 1.92%, SD: 1.74%; EtOH: 23.29%, SD: 35.59%) are the most abundant taxa. In EtOH-preserved adults, *Pluralibacter* (fresh: 0.15%, SD: 0.2%; EtOH: 61.41%, SD: 11.45%) was dominant, followed by *Acinetobacter* (fresh: 0.60%, SD: 1.03%; EtOH: 14.63%, SD: 21.2%), *Serratia* (fresh: 3.43%, SD: 5.9%; EtOH: 6.52%, SD: 7.02%), *Klebsiella* (fresh: 3.01%, SD: 6.36%; EtOH: 3.73%, SD: 1.87%), *Cronobacter* (fresh: 0.02%, SD: 0.03%; EtOH: 3.28%, SD: 2.14%), and *Enterobacter* (fresh: 0.01%, SD: 0.01%, EtOH: 2.96%, SD: 2.18%). Additionally, even if the qualitative composition of within group replicates was remarkably similar (particularly for the dominant groups, see Supplementary Table S10), quantitative differences and differences of low abundant genera could also be observed (see error bars of Figure 3). Details about the taxa relative abundance and variability are provided in Supplementary Table S10.

### Impact of Sample Manipulation Across Fruit Fly Samples From Different Sources

**FIGURE 4:**
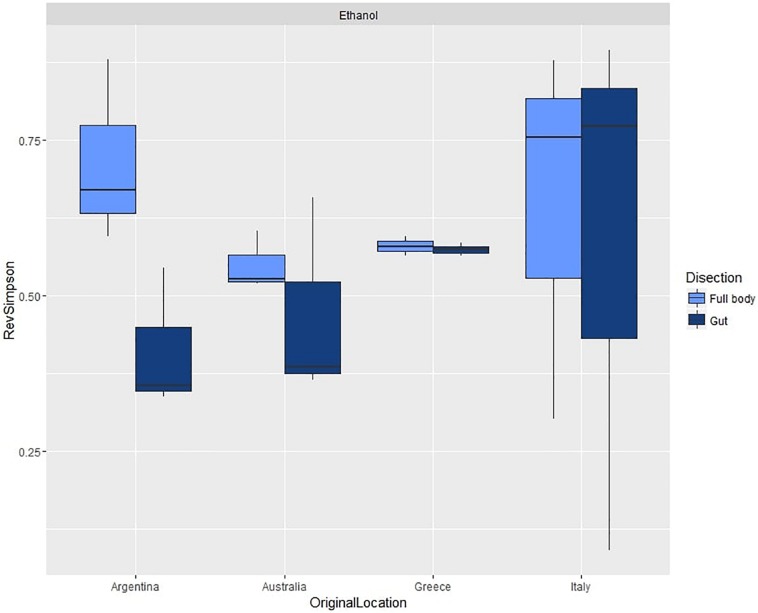
Box plots of average reverse Simpson indexes (+SD) calculated from gut and full body samples across different populations of *C. capitata*.

For the second experiment, we observed an average diversity per sample of 44.42 OTUs (SD = 19.62) and an average reverse Simpson index of 0.57 (SD = 0.20). ANOVA (Table 1B and Supplementary Table S7b) did not show significant differences in species diversity between dissection methods or across populations (Figure 4).

However, PERMANOVA (Table 1B and Supplementary Table S8b) did reveal significant variability across populations from different origin, with significant differences in all pairwise comparisons, while it did not detect differences between the multivariate patterns of dissected and non-dissected samples.

Similarly, PERMDISP (Table 1B and Supplementary Table S9b) showed highly significant differences across populations, with average within group dissimilarities ranging from 16.824 (Greece) to 76.975 (Argentina). Significant differences occurred in comparisons between all populations, except between the Italian and Argentinian population. PERMDISP also did not detect significant differences between dissected guts or full bodies.

**FIGURE 5:**
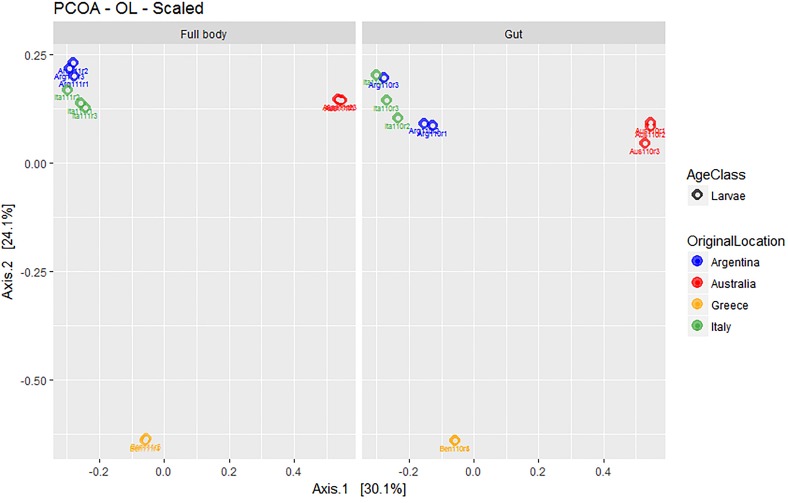
Multivariate ordination (PCOA) of gut microbial assemblages in ethanol preserved third instar larvae of *C. capitata* with different origin (colony – Greece, colony – Argentina, colony – Australia, wild – Italy). For clarity, the same PCOA is represented twice, either with full body **(left)** or gut-dissected samples **(right)** indicated. Colors represent original origin of samples (blue: Argentina, red: Australia, yellow: Greece, green: Italy).

Overall, the first two axes of the PCOA (Figure 5) explained 54.3% of variation (30.1 and 24.1% for PC1 and PC2, respectively). The visual analysis of Figure 5 further suggested the lack of relevant differences between the microbial assemblages obtained from full body and gut-dissected samples. Populations from Greece (with remarkably low dispersion around the corresponding group centroid) and Australia formed separate groups while the Argentinian and Italian populations grouped closer together.

**FIGURE 6:**
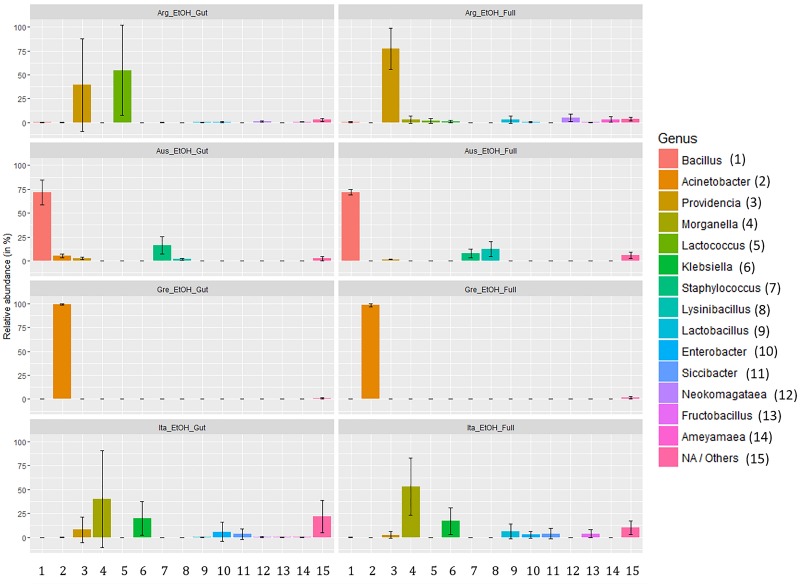
Relative abundance of gut symbionts (genus level classification) in ethanol preserved third instar larvae of *C. capitata* from different samples (colony – Greece, colony – Argentina, colony – Australia, wild – Italy).

Analyzing the taxon compositions across populations (Figure 6) further confirmed minor differences related to the dissection protocol. The microbiome compositions of abundant genera were remarkably similar in full body and gut samples with only some genera having major quantitative differences. The most notable of these are the genera *Lactococcus* (gut: 54.71%, SD: 47.32%; full body: 1.56%, SD: 2.58%) and *Providencia* (gut: 39.35%, SD: 48.34%; full body: 77.51%, SD: 41.41%) in the Argentinian population and *Lysinibacillus* (gut: 1.77%, SD: 1.08%; full body: 12.46%, SD: 7.7%) in the Australian population. Beside this, there are only differences in low abundance genera (details are provided in Supplementary Table S11). Conversely, we observed considerable variation across populations. Argentina samples were dominated by *Providencia* (58.43%) and *Lactobacillus* (28.13%) complemented with a number of genera in low abundance. Australian samples are dominated by *Bacillus* (71.91%) followed by *Staphylococcus* (11.94%) and *Lysinibacillus* (7.11%). Samples from Greece were dominated by *Acinetobacter* (98.63%). Italian samples had the most even spread, dominated by *Morganella* (46.59%), and *Klebsiella* (18.58%) but having multiple genera with a significant presence [*Providencia* (5.27%), *Enterobacter* (4.36%), *Lactobacillus* (3.30%), etc.]. Only a few genera, *Providencia*, *Acinetobacter*, *Morganella*, and *Klebsiella*, were presented across all populations. Details about the taxa relative abundance and variability are provided in Supplementary Table S11.

## Discussion

This study explored the effects of commonly used sample processing protocols on the gut microbiome of *C. capitata* recovered by amplicon sequencing of 16S *rRNA* genes across life stages and samples from different sources. Our results showed that the gut microbiome of the target *C. capitata* colonies mainly consisted of members of the Proteobacteria (>91% of reads) and Firmicutes, and to a lesser extent, Bacteroidetes and Actinobacteria. The phylum of Proteobacteria was mainly composed of members of the Enterobacteriaceae family. This composition is compatible with other studies done in *C. capitata* and other tephritid fruit flies (Behar et al., 2008b; Prabhakar et al., 2013; Andongma et al., 2015; Yong et al., 2016). Comparing microbiome composition at genus level, we find that most samples studied in this experiment are dominated by only one or two genera. Although there is considerable variation across samples, one of the most dominant bacterial genera was *Providencia* (Supplementary Table S2). This genus has been commonly detected as a part of the gut microbiome of *C. capitata* and other fruit fly species (Allwood and Drew, 1996; Behar et al., 2008a; Ami et al., 2010; Wang et al., 2011; Augustinos et al., 2015; IAEA, 2016). In contrast to many other members from the Enterobacteriaceae family, this genus consists mainly of opportunistic pathogenic species (Boemare et al., 1996; Juneja and Lazzaro, 2009; Galac and Lazzaro, 2011), so it would be interesting to investigate the potential role of *Providencia* in medfly and find out if it acts as a beneficial or parasitic partner. Other dominant genera included *Bacillus*, *Acinetobacter*, *Staphylococcus*, and *Morganella*. All of these genera have been recorded in multiple earlier studies of the fruit fly gut microbiome (Kuzina et al., 2001; Alma, 2008; Thaochan et al., 2010; Wang et al., 2011; Yuval et al., 2013; Hadapad et al., 2015; IAEA, 2016; Liu et al., 2016; Yong et al., 2016).

From our comparison of different sample treatment and preservation protocols, we can draw multiple conclusions regarding the microbiome composition. First of all, we found that dissection of the gut seems to have little impact on the microbiome profiling. Across all methods used to compare gut dissection against the use of full bodies (e.g., diversity indexes, PERMANOVA, PERMDISP, PCOA, and taxonomic composition), we only found a significant differences between gut and full body microbiome profiles in fresh samples from Experiment 1 (see Supplementary Table S8a). However, analysis of the PCOA (Figure 2) and visual inspection of taxon composition (see Supplementary Table S10) suggested that this difference was related to a lower relative abundance of the most dominant genus, *Providencia*, in full body samples. While there were only minor differences in relative abundance in many other genera. This results in similar patterns of composition to be found between gut dissection and use of full bodies. This relatively little difference observed between gut and full body microbiome profiles suggests that the gut bacterial community is predominant or outnumber bacteria occurring in the rest of the body and/or that bacterial communities end up mixing during dissection.

In contrast to the limited effect of dissection, preserving fruit flies in 70% EtOH strongly affected the microbiota composition as revealed through 16S *rRNA* gene amplicon sequencing. This is consistent with recent studies of the effects of EtOH preservation of different fecal samples (Hale et al., 2015; Sinha et al., 2016; Song et al., 2016). To our knowledge this is the first time the effects of 70% EtOH preservation have been shown in insect tissue. The most prominent effect found in this experiment was an increase in the evenness of EtOH-preserved samples, as indicated by the Simpson index, through the decrease in abundance of *Providencia*, i.e., the most dominant genus observed in fresh specimens. This was consistently observed in all different life stages and is reflected in the comparison of the reverse Simpson index (see Table 1A and Figure 1). Seeing we encountered *Providencia* in much lower relative abundance in all EtOH-preserved samples, we detected other genera with higher relative abundances. *Staphylococcus*, *Salinicoccus*, *Acinetobacter*, *Serratia*, and *Klebsiella*. All had a low abundance in fresh samples but dominate EtOH-preserved samples. Beside these major changes, we also found minor changes for many genera. For some, this was a small decrease in relative abundance for EtOH-preserved samples, while for others there was a small increase. For a number of genera, with a very low abundance, these small changes made the difference between being detected or not. It is likely that genera that have a decreased relative abundance in EtOH-preserved samples, such as *Providencia*, are negatively affected by EtOH preservation more than the other genera, while this decrease makes it easier for other genera to be detected. Hale et al. (2015) reported that in fecal samples preservation methods could exhibit this kind of bias toward or against certain microbiological groups. EtOH preservation does not only affect microbiome composition but also affects the variation between replicate samples.

In general, comparison of the different life stages showed that the gut microbiota of larvae is not only different in composition, but also have a lower diversity and variation when compared to adults and tenerals. This is not unexpected when taking in account the different feeding, lower mobility, and interaction with the environment of larvae in comparison with adults. However in larvae, we found that the multivariate dispersion in EtOH-preserved samples was much lower than in fresh samples. This is in contrast to what was found in tenerals and adults where fresh samples showed a lower dispersion. It is likely that this is linked to the complexity of the gut microbiome. In larvae, we found that after EtOH preservation samples are again completely dominated by one genus, *Acinetobacter*, while in tenerals and adults there is a more even spread with multiple genera being dominant. Additionally, in all EtOH-preserved samples differences in the relative abundances of a number of taxa could also be observed. The non-consistent patterns observed suggest that the use of 70% EtOH as preservative might produce unpredictable effects on the microbiome profiles of samples, including not consistent shifts in the relative proportion of the less abundant bacterial taxa. However, further experimental validation is necessary to verify this hypothesis.

In all fresh samples we found comparable microbiome compositions even across life stages, which were all consistently dominated by members of the genus *Providencia*. As expected, many differences can be found when looking at the less abundant bacterial taxa and this seems the reason of the difference in microbial profiles between fresh tenerals and adults (see Supplementary Table S8a). Conversely, in EtOH-preserved samples we detected major differences across the different life stages. We hypothesize that the reduced abundance *Providencia* after EtOH preservation allows less abundant taxa to dominate or become detectable in the gut microbiome profiles from EtOH-preserved samples. This, and the added unpredictability, might magnify the differences between life stages, and showing no consistent differences in gut microbiome composition. Therefore, our tests on EtOH-preserved specimens seem to confirm earlier studies that shifts in the gut microbiome profiles occur across fruit fly development stages (Aharon et al., 2013; Andongma et al., 2015). These results should be taken cautiously as compositional changes observed in fresh specimens seem to be far less impressive.

The second experiment allowed us to verify the consistency of patterns observed for the first experiments on an heterogeneous group of fruit fly samples from different sources. In this experiment we observed high variability in both diversity and composition of microbiome profiles (Table 1B). Even the Greek, Australian, and Argentinian laboratory populations, which were reared with an identical diet and similar environmental conditions, still had very distinct gut microbiome compositions. It is however difficult to speculate about the combination of processes responsible for the observed patterns. The geographical origin of samples seems to have a very strong effect on the gut microbiome composition; however, there also seemed that there is a correspondence between the colony age and the diversity within/variation between samples. The larvae from the (long established) Greek colony populations showed the lowest variation, followed by the (intermediately established) Australian colony population. The variation in the Italian (wild) population and (recently established) Argentinian colony population was much higher (see Supplementary Table S9b). This result seemed to be in line with results of previous studies comparing diversity and composition of the gut microbiome from colony and wild fruit fly populations (Tsiropoulos, 1983; Konstantopoulou et al., 1999; Ben-yosef et al., 2015; Morrow et al., 2015; Deutscher et al., 2018; Malacrinò et al., 2018). To adequately disentangle the effects of colony age and colony origin, targeted experiments with adequately replicated samples of similar ages and/or origins are needed.

## Conclusion

In conclusion, we found that differences in gut microbial profiles obtained from gut dissected and non-dissected samples were only minor with patterns that were stable across all life stage and samples from different sources. In contrast, preservation of samples in 70% EtOH had a major effect on the resulting gut microbiome profiles and was associated to higher inter-replicate variability and not consistent changes across life stages. These results shed new light on how samples preparation protocols can affect the results of HTS experiments and will help us interpreting and cross-compare the results of future and past studies. This study suggests that standardizing wet-lab procedures will increase the consistency, reliability, and repeatability of microbiomic research. A recommendation could be made against the use of 70% EtOH, a widely used preservatives in entomology, as the proportion of water is still probably too high to guarantee efficient gut microbiome fixation and preservation. When possible, fresh material or of more efficient preservative approaches (tentatively including deep freezing and absolute EtOH) should be preferred. Further experiments comparing different sample preparation protocols, different preservation techniques, on different model organisms, might give more insight in the use alternative sample preparation and preservation protocols. These studies will help us to further identify the effects of variations in the sample preparation and help pave the way a more comprehensive understanding of the insect gut microbiome.

## Data Availability Statement

The datasets generated for this study can be found in the NCBI Sequence Read Archive (https://www.ncbi.nlm.nih.gov/sra) and can be accessed with the accession numbers: SRR8732251–SRR8732302.

## Author Contributions

MD, AW, MV, and PV designed the research and secured the funding. MD, AA, KB, and MV designed and performed the experiments. MD and MV analyzed the data with input from all other authors. MD wrote the manuscript. All authors proofread, edited, and approved the manuscript.

## Conflict of Interest

The authors declare that the research was conducted in the absence of any commercial or financial relationships that could be construed as a potential conflict of interest.

## Footnotes

1 http://bccm.belspo.be/

## Supplementary Material

TABLE S1 Results of DNA barcoding identifications implemented on wild samples; identified species: Top matched species, Top%: highest similarity with the identified species, Low%: lowest similarity with the identified species.

TABLE S2 Overview of the experimental setup: Experiment 1: three life stages (larvae, tenerals, and adults), two forms of preservation (fresh and preserved in 70% ethanol), and two forms of samples preparation (dissected and full body) across one population of *C. capitata*. Experiment 2: one life stage (larvae), two forms of preservation (fresh and preserved in 70% ethanol), and two forms of samples preparation (dissected and full body) across three different colony strains and one wild population.

TABLE S3 List of the considered *C. capitata* samples for this study.

TABLE S4 Detailed analytical pipeline implemented for data filtering and taxon assignment.

TABLE S5 Overview of sequencing data and taxon assignment after data filtering.

TABLE S6 Relative composition of groups in all samples of *C. capitata* included in this study. Inner circle: phylum composition; outer circle: most abundant genera.

TABLE S7 ANOVA and *a posteriori* comparisons (Tukey’s HSD test) testing for differences in patterns of alpha diversity (as estimated by the Reverse Simpson index calculated from OTU data) across **(A)** dissection procedures, sample preservation methods, and life stages of *C. capitata* and **(B)** dissection procedures on different populations of *C. capitata*. df, degrees of freedom; MS, mean square estimates; F, pseudo-*F*; P, *p*-value; n.s., not significant; and ^∗∗∗^, at *P* < 0.001. Tukey’s HSD test: diff, difference in test scores; lwr–upr, lower and upper limits of 95% confidence interval; *p*-adj, false discovery rate-adjusted *p*-value.

TABLE S8 PERMANOVA and *a posteriori* comparisons (*t*-tests) verifying differences in OTUs abundance and composition across **(A)** dissection procedures, sample preservation methods, and life stages of *C. capitata* and **(B)** dissection procedures on different populations of *C. capitata*. df, degrees of freedom; MS, mean square estimates; F, pseudo-*F*; P, *p*-value; n.s., not significant; and ^∗^, significant at *P* < 0.05, ^∗∗^, at *P* < 0.01, ^∗∗∗^, at *P* < 0.001.

TABLE S9 PERMDISP and *a posteriori* comparisons (*t*-tests) verifying differences in variation within samples across **(A)** sample preservation methods and life stages of *C. capitata* and **(B)** dissection procedures on different populations of *C. capitata*. df, degrees of freedom; MS, mean square estimates; F, pseudo-*F*; P, *p*-value; n.s., not significant; and ^∗^, significant at *P* < 0.05, ^∗∗^, at *P* < 0.01, ^∗∗∗^, at *P* < 0.001.

TABLE S10 Overview of average relative abundance (+SD) of the 15 most abundant genera in fresh and ethanol preserved specimens of reared larvae, teneral, and adults from the Greek laboratory colony of *C. capitata*.

TABLE S11 Overview of average relative abundance (+SD) of the 15 most abundant genera in fresh and ethanol preserved third instar larvae of *C. capitata* with different origin.
