# Supplementary material for: Impact of Sample Preservation and Manipulation on Insect Gut Microbiome Profiling. A Test Case With Fruit Flies (Diptera, Tephritidae)
Source: Front Microbiol. 2019 Dec 13;10:2833. doi: 10.3389/fmicb.2019.02833 (PMC6923184; doi:10.3389/fmicb.2019.02833)
Supplement: TABLE S2 — Overview of the experimental setup: Experiment 1: three life stages (larvae, tenerals, and adults), two forms of preservation (fresh and preserved in 70% ethanol), and two forms of samples preparation (dissected and full body) across one population of C. capitata. Experiment 2: one life stage (larvae), two forms of preservation (fresh and preserved in 70% ethanol), and two forms of samples preparation (dissected and full body) across three different colony strains and one wild population. [file Table_2.DOCX]

Supplementary Material

***SI 2. Overview of the experimental setup****: Experiment 1: three life stages (Larvae, Tenerals, Adults), two forms of preservation (Fresh, Preserved in 70% ethanol) and two forms of samples preparation (Dissected, Full body) across one population of C. capitata. Experiment 2: one life stage (Larvae), two forms of preservation (Fresh, Preserved in 70% ethanol) and two forms of samples preparation (Dissected, Full body) across three different colony strains and one wild population.*

Experiment 1:

| Age | Larvae | | | | Tenerals | | | | Adults | | | |
| --- | --- | --- | --- | --- | --- | --- | --- | --- | --- | --- | --- | --- |
| Preservation | Fresh | | Ethanol | | Fresh | | Ethanol | | Fresh | | Ethanol | |
| Dissection | Gut | Full | Gut | Full | Gut | Full | Gut | Full | Gut | Full | Gut | Full |
| Greek pop. | **3 rep.** | **3 rep.** | **3 rep.** | **3 rep.** | **3 rep.** | **3 rep.** | **3 rep.** | **3 rep.** | **3 rep.** | **3 rep.** | **3 rep.** | **3 rep.** |

Experiment 2:

| Age | Larvae | |
| --- | --- | --- |
| Preservation | Ethanol preserved | |
| Dissection | Gut | Full Body |
| Greek pop. | **3 replicates** | **3 replicates** |
| Australian pop. | **3 replicates** | **3 replicates** |
| Argentinia pop. | **3 replicates** | **3 replicates** |
| Italian pop. | **3 replicates** | **3 replicates** |
