# Supplementary material for: Impact of Sample Preservation and Manipulation on Insect Gut Microbiome Profiling. A Test Case With Fruit Flies (Diptera, Tephritidae)
Source: Front Microbiol. 2019 Dec 13;10:2833. doi: 10.3389/fmicb.2019.02833 (PMC6923184; doi:10.3389/fmicb.2019.02833)
Supplement: TABLE S3 — List of the considered C. capitata samples for this study. [file Table_3.DOCX]

Supplementary Material

***SI 3. List of the considered C. capitata samples for this study***

| Collection location | Original location | Life stage | Collection date | Preservation | Processing date |
| --- | --- | --- | --- | --- | --- |
| IPCL (Seibersdorf), Austria | Greece | Larvae | aug/16 | Fresh | aug/16 |
| IPCL (Seibersdorf), Austria | Greece | Larvae | aug/16 | Ethanol | nov/16 |
| IPCL (Seibersdorf), Austria | Greece | Teneral | aug/16 | Fresh | aug/16 |
| IPCL (Seibersdorf), Austria | Greece | Teneral | aug/16 | Ethanol | nov/16 |
| IPCL (Seibersdorf), Austria | Greece | Adult | aug/16 | Fresh | aug/16 |
| IPCL (Seibersdorf), Austria | Greece | Adult | aug/16 | Ethanol | nov/16 |
| IPCL (Seibersdorf), Austria | Australia | Larvae | aug/16 | Ethanol | nov/16 |
| IPCL (Seibersdorf), Austria | Argentina | Larvae | sep/16 | Ethanol | dec/16 |
| zio Augusto, Italy | Italy | Larvae | aug/16 | Ethanol | nov/16 |
