# Supplementary material for: Impact of Sample Preservation and Manipulation on Insect Gut Microbiome Profiling. A Test Case With Fruit Flies (Diptera, Tephritidae)
Source: Front Microbiol. 2019 Dec 13;10:2833. doi: 10.3389/fmicb.2019.02833 (PMC6923184; doi:10.3389/fmicb.2019.02833)
Supplement: TABLE S4 — Detailed analytical pipeline implemented for data filtering and taxon assignment. [file Table_4.DOCX]

Supplementary Material

***SI 4. Detailed analytical pipeline implemented for data filtering and taxon assignment***

# **Prepeartoin** #

library(dada2)

path <- "./MiSeqData"

list.files(path)

fnFs <- sort(list.files(path, pattern="_1.fastq", full.names = TRUE))

fnRs <- sort(list.files(path, pattern="_2.fastq", full.names = TRUE))

# Getting the samples namens (assuming name is structured : "SampleName_XXX.fastq)

sample.names <- sapply(strsplit(basename(fnFs), "_"), `[`, 1)

# **Trimming & Filtering** #

plotQualityProfile(fnFs[1:2])

plotQualityProfile(fnRs[1:2])

path <- "."

filt_path <- file.path(path, "filtered") # Place filtered files in filtered/ subdirectory

filtFs <- file.path(filt_path, paste0(sample.names, "_F_filt.fastq.gz"))

filtRs <- file.path(filt_path, paste0(sample.names, "_R_filt.fastq.gz"))

out <- filterAndTrim(fnFs, filtFs, fnRs, filtRs, truncLen=c(290,231),

maxN=0, maxEE=c(1,1), truncQ=1, trimLeft = 20, rm.phix=TRUE,

compress=TRUE, multithread=TRUE, verbose = TRUE)

# **Learing error rates** #

errF <- learnErrors(filtFs, multithread=TRUE,nreads = 10e+06)

errR <- learnErrors(filtRs, multithread=TRUE,nreads = 10e+06)

plotErrors(errF, nominalQ=TRUE)

plotErrors(errR, nominalQ=TRUE)

# **Dereplicate** #

derepFs <- derepFastq(filtFs, verbose=TRUE)

derepRs <- derepFastq(filtRs, verbose=TRUE)

names(derepFs) <- sample.names

names(derepRs) <- sample.names

# **Sample inference** #

dadaFs <- dada(derepFs, err=errF, multithread=TRUE)

dadaRs <- dada(derepRs, err=errR, multithread=TRUE)

# **Merge Pairends** #

mergers <- mergePairs(dadaFs, derepFs, dadaRs, derepRs, verbose=TRUE)

head(mergers[[1]])

# **Construct sequence table** #

seqtab <- makeSequenceTable(mergers)

dim(seqtab)

table(nchar(getSequences(seqtab)))

# If length is much smaller or lower then target remove using : seqtab2 <- seqtab[,nchar(colnames(seqtab)) %in% seq(415,500)]

seqtab2 <- seqtab[,nchar(colnames(seqtab)) %in% seq(415,500)]

dim(seqtab2)

table(nchar(getSequences(seqtab2)))

# **Remove Chimaera's** #

seqtab.nochim <- removeBimeraDenovo(seqtab2, method="consensus", multithread=TRUE, verbose=TRUE)

dim(seqtab.nochim)

sum(seqtab.nochim)/sum(seqtab)

write.csv(seqtab.nochim, file ="SEQTab.csv")

# **Track changes trough the pipeline** #

getN <- function(x) sum(getUniques(x))

track <- cbind(out, sapply(dadaFs, getN), sapply(mergers, getN), rowSums(seqtab), rowSums(seqtab.nochim))

colnames(track) <- c("input", "filtered", "denoised", "merged", "tabled", "nonchim")

rownames(track) <- sample.names

track

write.csv(track, file ="Track.csv")

# **Assign Taxomony** #

taxa <- assignTaxonomy(seqtab.nochim, "DataBases/Silva/silva_nr_v132_train_set.fa.gz", multithread=TRUE)

taxa <- addSpecies(taxa, "DataBases/Silva/silva_species_assignment_v132.fa.gz")

taxa.print <- taxa # Removing sequence rownames for display only

rownames(taxa.print) <- NULL

head(taxa.print)

write(taxa, file = "TaxonomicFile.csv")

# **Asses error rate** #

unqs.mock <- seqtab.nochim["Mock",]

unqs.mock <- sort(unqs.mock[unqs.mock>0], decreasing=TRUE) # Drop ASVs absent in the Mock

cat("DADA2 inferred", length(unqs.mock), "sample sequences present in the Mock community.\n")

mock.ref <- getSequences(file.path(path, "MockFasta.fasta"))

match.ref <- sum(sapply(names(unqs.mock), function(x) any(grepl(x, mock.ref))))

cat("Of those,", sum(match.ref), "were exact matches to the expected reference sequences.\n")

write(length(unqs.mock),file ="NumbMock.txt")

write(sum(match.ref), file ="MatchMock.txt")
