# Supplementary material for: Impact of Sample Preservation and Manipulation on Insect Gut Microbiome Profiling. A Test Case With Fruit Flies (Diptera, Tephritidae)
Source: Front Microbiol. 2019 Dec 13;10:2833. doi: 10.3389/fmicb.2019.02833 (PMC6923184; doi:10.3389/fmicb.2019.02833)

Supplementary Material

***SI 6****. Relative composition of groups in all samples of C. capitata included in this study. Inner circle: phylum composition; outer circle: most abundant genera*


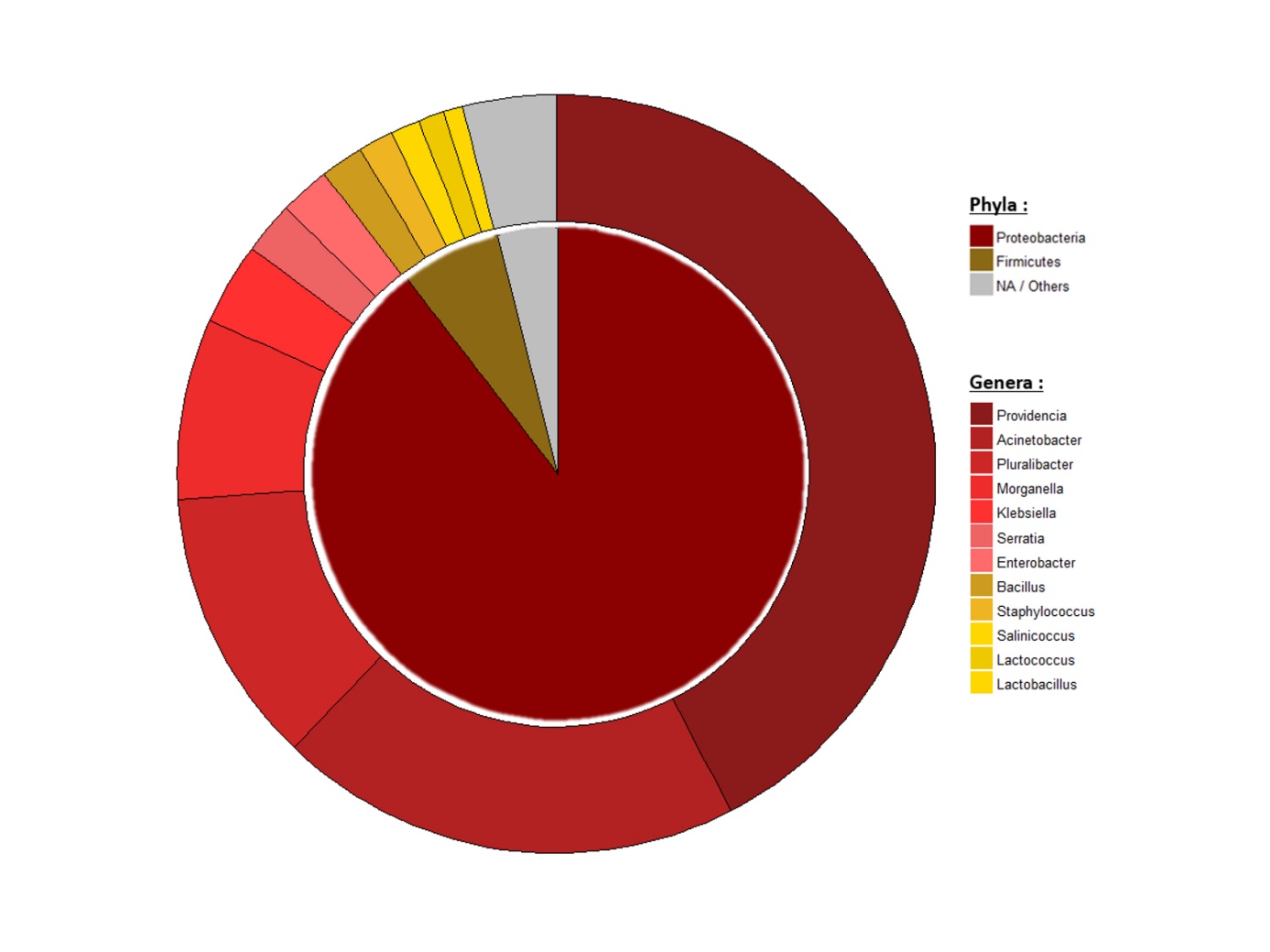

Supplement: TABLE S6 — Relative composition of groups in all samples of C. capitata included in this study. Inner circle: phylum composition; outer circle: most abundant genera. [file Table_6.docx]
