# Supplementary material for: Impact of Sample Preservation and Manipulation on Insect Gut Microbiome Profiling. A Test Case With Fruit Flies (Diptera, Tephritidae)
Source: Front Microbiol. 2019 Dec 13;10:2833. doi: 10.3389/fmicb.2019.02833 (PMC6923184; doi:10.3389/fmicb.2019.02833)
Supplement: TABLE S7 — ANOVA and a posteriori comparisons (Tukey’s HSD test) testing for differences in patterns of alpha diversity (as estimated by the Reverse Simpson index calculated from OTU data) across (A) dissection procedures, sample preservation methods, and life stages of C. capitata and (B) dissection procedures on different populations of C. capitata. df, degrees of freedom; MS, mean square estimates; F, pseudo-F; P, p-value; n.s., not significant; and ∗∗∗, at P < 0.001. Tukey’s HSD test: diff, difference in test scores; lwr–upr, lower and upper limits of 95% confidence interval; p-adj, false discovery rate-adjusted p-value. [file Table_7.DOCX]

Supplementary Material

***SI 7.*** *ANOVA and a posteriori comparisons (Tukey's HSD test) testing for differences in patterns of alpha diversity (as estimated by the Reverse Simpson index calculated from OTU data) across (a) dissection procedures, sample preservation methods and life stages of C. capitata and (b) dissection procedures on different populations of C. capitata. df: degrees of freedom; MS: mean square estimates; F: pseudo-F; P: p-value; n.s.: not significant, *: significant at P<0.05; **: at P<0.01, ***: at P<0.001. Tukey HSD test: diff: difference in test scores; lwr - upr: lower and upper limits of 95% confidence interval; p adj: false discovery rate adjusted p-value*

| Reverse Simpson Index – OTUs **(a)** | df | MS | F | P |  |
| --- | --- | --- | --- | --- | --- |
| Life stage (li) | 2 | 0.072 | 1.621 | 0.221 | *n.s.* |
| Preservation (pr) | 1 | 0.828 | 18.801 | 0.000 | *** |
| Disection (di) | 1 | 0.121 | 2.742 | 0.112 | *n.s.* |
| Li x pr | 2 | 0.040 | 0.899 | 0.421 | *n.s.* |
| Li x di | 2 | 0.007 | 0.150 | 0.861 | *n.s.* |
| Pr x di | 1 | 0.118 | 2.674 | 0.116 | *n.s.* |
| Li x pr x di | 2 | 0.000 | 0.009 | 0.991 | *n.s.* |
| Residual | 22 | 0.044 |  |  |  |
|  |  |  |  |  |  |
| **Tukey HSD : Preservation** | diff | lwr | upr | p adj |  |
| Fresh - Ethanol | -0.312 | -0.462 | -0.162 | 0.000 | *** |

| Reverse Simpson Index – OTUs **(b)** | df | MS | F | P |  |
| --- | --- | --- | --- | --- | --- |
| Origin (Or) | 3 | 0.011 | 0.232 | 0.873 | n.s. |
| Dissection (Di) | 1 | 0.081 | 1.658 | 0.219 | n.s. |
| Ol x Di | 3 | 0.024 | 0.481 | 0.701 | n.s. |
| Residuals | 14 | 0.049 |  |  |  |
