# Supplementary material for: Impact of Sample Preservation and Manipulation on Insect Gut Microbiome Profiling. A Test Case With Fruit Flies (Diptera, Tephritidae)
Source: Front Microbiol. 2019 Dec 13;10:2833. doi: 10.3389/fmicb.2019.02833 (PMC6923184; doi:10.3389/fmicb.2019.02833)
Supplement: TABLE S8 — PERMANOVA and a posteriori comparisons (t-tests) verifying differences in OTUs abundance and composition across (A) dissection procedures, sample preservation methods, and life stages of C. capitata and (B) dissection procedures on different populations of C. capitata. df, degrees of freedom; MS, mean square estimates; F, pseudo-F; P, p-value; n.s., not significant; and ∗, significant at P < 0.05, ∗∗, at P < 0.01, ∗∗∗, at P < 0.001. [file Table_8.DOCX]

Supplementary Material

***SI 8.*** *PERMANOVA and a posteriori comparisons (t-tests) verifying differences in OTUs abundance and composition across (a) dissection procedures, sample preservation methods and life stages of C. capitata and (b) dissection procedures on different populations of C. capitata. df, degrees of freedom; MS: mean square estimates; F: pseudo-F; P: p-value; n.s.: not significant, *: significant at P<0.05; **: at P<0.01, ***: at P<0.001*

| **(a)** | df | MS | F | P |  |
| --- | --- | --- | --- | --- | --- |
| Life stage (li) | 2 | 12922.647 | 23.941 | 0.000 | *** |
| Preservation (pr) | 1 | 38101.057 | 70.587 | 0.000 | *** |
| Dissection (di) | 1 | 864.028 | 1.601 | 0.175 | n.s. |
| Li x pr | 2 | 12561.108 | 23.271 | 0.000 | *** |
| Li x di | 2 | 829.980 | 1.538 | 0.161 | n.s. |
| Pr x di | 1 | 1450.149 | 2.687 | 0.047 | * |
| Li x pr x di | 2 | 856.388 | 1.587 | 0.147 | n.s. |
| Residual | 24 | 539.778 |  |  |  |
|  |  |  |  |  |  |
| **Post-Hoc : Life stage x Preservation** | t | P |  |  |  |
| Preservation : **Fresh** |  |  |  |  |  |
| Larvae - Teneral | 1.377 | 0.137 | n.s. |  |  |
| Larvae - Adult | 1.484 | 0.099 | n.s. |  |  |
| Teneral - Adult | 1.624 | 0.037 | * |  |  |
|  |  |  |  |  |  |
| Preservation : **Ethanol** |  |  |  |  |  |
| Larvae - Teneral | 4.734 | 0.002 | ** |  |  |
| Larvae - Adult | 9.661 | 0.002 | ** |  |  |
| Teneral - Adult | 3.952 | 0.002 | ** |  |  |
|  |  |  |  |  |  |
| **Post-Hoc : Preservation x Dissection** | t | P |  |  |  |
| Preservation : **Fresh** |  |  |  |  |  |
| Gut - Full body | 1.567 | 0.040 | * |  |  |
|  |  |  |  |  |  |
| Preservation : **Ethanol** |  |  |  |  |  |
| Gut - Full body | 0.620 | 0.854 | n.s. |  |  |

| **(b)** | df | MS | F | P |  |
| --- | --- | --- | --- | --- | --- |
| Origin (or) | 3 | 22846.349 | 21.592 | 0.000 | *** |
| Dissection (di) | 1 | 1568.855 | 0.882 | 0.657 | n.s. |
| Or x di | 3 | 1779.535 | 1.682 | 0.068 | n.s. |
| Residual | 16 | 1058.112 |  |  |  |
|  |  |  |  |  |  |
| **Post-Hoc : Origin** | t | P |  |  |  |
| Argentina vs. Australia | 4.147 | 0.002 | ** |  |  |
| Argentina vs. Greece | 4.567 | 0.002 | ** |  |  |
| Argentina vs. Italy | 2.619 | 0.002 | ** |  |  |
| Australia vs. Greece | 13.862 | 0.002 | ** |  |  |
| Australia vs. Italy | 4.351 | 0.002 | ** |  |  |
| Greece vs. Italy | 4.742 | 0.002 | ** |  |  |
