# Supplementary material for: Impact of Sample Preservation and Manipulation on Insect Gut Microbiome Profiling. A Test Case With Fruit Flies (Diptera, Tephritidae)
Source: Front Microbiol. 2019 Dec 13;10:2833. doi: 10.3389/fmicb.2019.02833 (PMC6923184; doi:10.3389/fmicb.2019.02833)
Supplement: TABLE S9 — PERMDISP and a posteriori comparisons (t-tests) verifying differences in variation within samples across (A) sample preservation methods and life stages of C. capitata and (B) dissection procedures on different populations of C. capitata. df, degrees of freedom; MS, mean square estimates; F, pseudo-F; P, p-value; n.s., not significant; and ∗, significant at P < 0.05, ∗∗, at P < 0.01, ∗∗∗, at P < 0.001. [file Table_9.DOCX]

Supplementary Material

***SI 9.*** *PERMDISP and a posteriori comparisons (t-tests) verifying differences in variation within samples across (a) sample preservation methods and life stages of C. capitata and (b) dissection procedures on different populations of C. capitata. df, degrees of freedom; MS: mean square estimates; F: pseudo-F; P: p-value; n.s.: not significant, *: significant at P<0.05; **: at P<0.01, ***: at P<0.001*

| **(a)** | df | | MS | | | | | F | | | | P | | | |  | | |  |  |  |
| --- | --- | --- | --- | --- | --- | --- | --- | --- | --- | --- | --- | --- | --- | --- | --- | --- | --- | --- | --- | --- | --- |
| Life stage (li) | 2 | | 1572.520 | | | | | 2.054 | | | | 0.325 | | | | n.s. | | |  |  |  |
| Preservation (pr) | 1 | | 447.947 | | | | | 0.585 | | | | 0.527 | | | | n.s. | | |  |  |  |
| Li x pr | 2 | | 765.660 | | | | | 7.984 | | | | 0.001 | | | | *** | | |  |  |  |
| Resid | 30 | | 95.895 | | | | |  | | | |  | | | |  | | |  |  |  |
|  |  | |  | | | | |  | | | |  | | | |  |  |  |  |  |  |
| **Post Hoc: Life stage x Preservation** | t | | P | | | | |  | | | |  | | | |  |  |  |  |  |  |
| Preservation: **Fresh** |  | |  | | | | |  | | | |  | | | |  |  |  |  |  |  |
| Larvae - Teneral | 1.223 | | 0.269 | | | | | n.s. | | | |  | | | |  |  |  |  |  |  |
| Larvae - Adult | 0.641 | | 0.517 | | | | | n.s. | | | |  | | | |  |  |  |  |  |  |
| Teneral - Adult | 0.722 | | 0.506 | | | | | n.s. | | | |  | | | |  |  |  |  |  |  |
|  |  | |  | | | | |  | | | |  | | | |  |  |  |  |  |  |
| Preservation: **Ethanol** |  | |  | | | | |  | | | |  | | | |  |  |  |  |  |  |
| Larvae - Teneral | 6.333 | | 0.002 | | | | | ** | | | |  | | | |  |  |  |  |  |  |
| Larvae - Adult | 4.699 | | 0.002 | | | | | ** | | | |  | | | |  |  |  |  |  |  |
| Teneral - Adult | 2.680 | | 0.018 | | | | | * | | | |  | | | |  |  |  |  |  |  |
|  |  | |  | | |  | | | |  | | | |  | | | |  | | |  |
| **Average within group dissimilarities** |  | |  | | |  | | | |  | | | |  | | | |  | | |  |
| \| **Fresh** \|  \|  \| \| --- \| --- \| --- \| \|  \| Larvae \| 14.717 \| \|  \| Teneral \| 26.374 \| \|  \| Adult \| 19.654 \| \|  \|  \|  \| \| **Ethanol** \|  \|  \| \|  \| Larvae \| 2.091 \| \|  \| Teneral \| 58.335 \| \|  \| Adult \| 31.031 \| \|  \|  \|  \| |  | |  | |  | | | |  | | | |  |  |  |  |  |  |  |  |  |
| **(b)** | | df | | MS | | | F | | | | P | | | |  | | | | |  |  |
| Origin (or) | | 3 | | 1750.133 | | | 15.948 | | | | 0.025 | | | | * | | | | |  |  |
| Dissection (di) | | 1 | | 438.219 | | | 3.993 | | | | 0.140 | | | | n.s. | | | | |  |  |
| Or x di | | 3 | | 109.741 | | | 1.338 | | | | 0.296 | | | | n.s. | | | | |  |  |
| Resid | | 16 | | 82.016 | | |  | | | |  | | | |  | | | | |  |  |
|  | |  | |  | | |  | | | |  | | | |  | | | | |  |  |
| **Post Hoc: Origin** | | t | | P | | |  | | | |  | | | |  | | | | |  |  |
| Argentina vs. Australia | | 3.056 | | 0.005 | | | ** | | | |  | | | |  | | | | |  |  |
| Argentina vs. Greece | | 4.657 | | 0.002 | | | ** | | | |  | | | |  | | | | |  |  |
| Argentina vs. Italy | | 0.862 | | 0.401 | | | *n.s.* | | | |  | | | |  | | | | |  |  |
| Australia vs. Greece | | 5.651 | | 0.002 | | | * | | | |  | | | |  | | | | |  |  |
| Australia vs. Italy | | 5.046 | | 0.002 | | | ** | | | |  | | | |  | | | | |  |  |
| Greece vs. Italy | | 7.162 | | 0.002 | | | ** | | | |  | | | |  | | | | |  |  |
|  | |  | |  | | |  | | | |  | | | |  | | | | |  |  |
| **Average within group dissimilarities** | | | | | | |  | | | |  | | | |  | | | | |  |  |
| Argentina | | 59.766 | |  | | |  | | | |  | | | |  | | | | |  |  |
| Australia | | 22.424 | |  | | |  | | | |  | | | |  | | | | |  |  |
| Greece | | 2.091 | |  | | |  | | | |  | | | |  | | | | |  |  |
| Italy | | 62.554 | |  | | |  | | | |  | | | |  | | | | |  |  |
