# Supplementary material for: Impact of Sample Preservation and Manipulation on Insect Gut Microbiome Profiling. A Test Case With Fruit Flies (Diptera, Tephritidae)
Source: Front Microbiol. 2019 Dec 13;10:2833. doi: 10.3389/fmicb.2019.02833 (PMC6923184; doi:10.3389/fmicb.2019.02833)
Supplement: TABLE S10 — Overview of average relative abundance (+SD) of the 15 most abundant genera in fresh and ethanol preserved specimens of reared larvae, teneral, and adults from the Greek laboratory colony of C. capitata. [file Table_10.DOCX]

Supplementary Material

***SI 10. Overview of average relative abundance (+SD) of the 15 most abundant genera in fresh and ethanol preserved specimens of reared larvae, teneral and adults from the Greek laboratory colony of C. capitata.***

| Genus  (# OTUs) | Gre_Larvae  _Fresh | Gre_Larvae  _EtOH | Gre_Teneral  _Fresh | Gre_Teneral  _EtOH | Gre_Adult  _Fresh | Gre_Adult  _EtOH |
| --- | --- | --- | --- | --- | --- | --- |
| Providencia  (54) | 92.57  [12.80] | 0.14  [0.19] | 80.66  [19.27] | 35.83  [35.82] | 85.22  [15.04] | 0.90  [0.96] |
| Acinetobacter  (42) | 0.05  [0.05] | 98.63  [1.11] | 0.13  [0.18] | 0.19  [0.22] | 0.60  [1.04] | 14.63  [21.20] |
| Pluralibacter  (19) | 3.41  [6.77] | 0.06  [0.11] | 2.95  [2.96] | 1.12  [0.69] | 0.15  [0.2] | 61.42  [11.45] |
| Salinicoccus  (18) | 0.00  [0.01] | 0.06  [0.12] | 1.35  [1.10] | 32.6  [27.23] | 0.00  [0.01] | 0.07  [0.09] |
| Staphylococcus  (14) | 0.18  [0.22] | 0.02  [0.01] | 1.92  [1.74] | 23.29  [35.59] | 0.03  [0.03] | 0.08  [0.04] |
| Serratia  (14) | 0  [0.01] | - | 0.06  [0.06] | 0.04  [0.03] | 3.43  [5.89] | 6.52  [7.07] |
| Klebsiella  (21) | 0.03  [0.07] | 0.02  [0.03] | 0.69  [1.54] | 0.07  [0.09] | 3.01  [6.36] | 3.73  [1.87] |
| Nesterenkonia  (9) | - | 0.01  [0.02] | 0.27  [0.29] | 3.71  [3.36] | - | 0.01  [0.03] |
| Cronobacter  (1) | - | - | - | 0.00  [0.01] | 0.02  [0.03] | 3.28  [2.14] |
| Enterobacter  (12) | - | 0.01  [0.02] | - | 0.02  [0.04] | 0.01  [0.01] | 2.69  [2.08] |
| Sphingobacterium  (12) | - | - | 1.64  [1.38] | 0.27  [0.19] | 0.02  [0.03] | 0.01  [0.02] |
| Siccibacter  (3) | - | - | - | - | 0.01  [0.02] | 1.85  [1.22] |
| Leuconostoc  (2) | - | 0.00  [0.01] | 1.50  [3.43] | 0.01  [0.01] | - | - |
| Erwinia  (2) | - | - | - | - | - | 1.36  [0.75] |
| NA / Others | 3.76  [5.98] | 1.04  [0.73] | 8.83  [9.73] | 2.85  [1.88] | 7.49  [6.56] | 3.45  [1.57] |
