# Supplementary material for: Impact of Sample Preservation and Manipulation on Insect Gut Microbiome Profiling. A Test Case With Fruit Flies (Diptera, Tephritidae)
Source: Front Microbiol. 2019 Dec 13;10:2833. doi: 10.3389/fmicb.2019.02833 (PMC6923184; doi:10.3389/fmicb.2019.02833)
Supplement: TABLE S11 — Overview of average relative abundance (+SD) of the 15 most abundant genera in fresh and ethanol preserved third instar larvae of C. capitata with different origin. [file Table_11.DOCX]

Supplementary Material

***SI 11. Overview of average relative abundance (+SD) of the 15 most abundant genera in fresh and ethanol preserved third instar larvae of C. capitata with different origin***

| Genus  (# OTUs) | Arg_Larvae  _EtOH_Gut | Arg_ Larvae  _EtOH_Full | Aus_ Larvae  _EtOH_Gut | Aus_ Larvae  _EtOH_Full | Gre_ Larvae  _EtOH_Gut | Gre_ Larvae  _EtOH_Full | Ita_ Larvae  _EtOH_Gut | Ita_ Larvae  _EtOH_Full |
| --- | --- | --- | --- | --- | --- | --- | --- | --- |
| Bacillus  (28) | 0.24  [0.13] | 0.45  [0.54] | 71.88  [12.93] | 71.95  [3.18] | - | - | 0.01  [0.01] | 0.02  [0.04] |
| Acinetobacter  (42) | 0.15  [0.10] | 0.01  [0.01] | 5.30  [1.81] | 0.12  [0.07] | 98.99  [0.74] | 98.27  [1.62] | 0.03  [0.03] | 0.02  [0.02] |
| Providencia  (54) | 39.35  [48.34] | 77.51  [21.45] | 2.49  [1.40] | 1.54  [0.32] | 0.04  [0.00] | 0.23  [0.26] | 7.98  [13.19] | 2.57  [3.66] |
| Morganella  (18) | 0.07  [0.04] | 3.10  [3.74] | 0.04  [0.07] | 0.07  [0.12] | 0.01  [0.01] | 0.00  [0.01] | 40.15  [50.76] | 53.04  [30.05] |
| Lactococcus  (8) | 54.71  [47.33] | 1.56  [2.56] | 0.03  [0.04] | 0.22  [0.09] | - | 0.01  [0.00] | 0.02  [0.02] | - |
| Klebsiella  (21) | 0.06  [0.09] | 1.16  [1.49] | 0.05  [0.09] | 0.04  [0.04] | 0.02  [0.01] | 0.03  [0.04] | 20.07  [17.52] | 17.09  [14.13] |
| Staphylococcus  (14) | 0.12  [0.10] | 0.02  [0.03] | 16.11  [9.16] | 7.77  [4.44] | 0.01  [0.01] | 0.02  [0.01] | - | 0.01  [0.01] |
| Lysinibacillus  (5) | 0.04  [0.04] | 0.02  [0.03] | 1.77  [1.08] | 12.46 [7.70] | - | - | - | - |
| Lactobacillus  (14) | 0.37  [0.28] | 3.07  [4.09] | - | 0.01  [0.01] | - | - | 0.13  [0.20] | 6.47  [7.80] |
| Enterobacter  (12) | 0.50  [0.75] | 0.48  [0.59] | - | 0.05  [0.04] | 0.00  [0.01] | 0.02  [0.03] | 5.89  [10.00] | 2.82  [3.63] |
| Siccibacter  (3) | - | - | - | - | - | - | 3.46  [5.62] | 3.95  [5.44] |
| Neokomagataea  (10) | 0.92  [0.61] | 5.04  [3.75] | 0.00  [0.01] | 0.01  [0.01] | - | - | 0.31  [0.53] | - |
| Fructobacillus  (7) | 0.01  [0.01] | 0.34  [0.31] | - | - | - | - | 0.25  [0.42] | 3.90  [4.37] |
| Ameyamaea  (22) | 0.59  [0.31] | 3.28  [3.08] | - | - | - | - | 0.09  [0.15] | 0.00  [0.01] |
| NA / Others | 2.87  [1.84] | 3.96  [1.85] | 2.32  [2.35] | 5.77  [3.38] | 0.93  [0.71] | 1.41  [1.39] | 21.62  [16.86] | 10.12  [7.00] |
